# BioRisk-S (Biological Risk–Stomatognathic): A Predictive Algorithm for Early Systemic Detection of Stomatognathic Dysfunction

**DOI:** 10.3390/bioengineering12121365

**Published:** 2025-12-16

**Authors:** Loredana Liliana Hurjui, Liliana Sachelarie, Carmen Stadoleanu, Rodica Maria Murineanu, Mircea Grigorian, Ioana Scrobota, Corina Laura Stefanescu

**Affiliations:** 1 Faculty of Medicine, Grigore T. Popa University of Medicine and Pharmacy Iasi, 16 Universitatii Str., 700115 Iasi, Romania; loredana.hurjui@umfiasi.ro; 2 Department of Dental Medicine, Apollonia University, 700511 Iasi, Romania; 3 Faculty of Medicine and Pharmacy, University Ovidius, Mamaia Boulevard, 900527 Constanta, Romaniamirceagrigorian@yahoo.com (M.G.);; 4 Department of Dental Medicine, Faculty of Medicine and Pharmacy, University of Oradea, 1 University Street, 410073 Oradea, Romania

**Keywords:** stomatognathic system, predictive algorithm, hs-CRP, NLR, vitamin D, BioRisk-S

## Abstract

Background: Functional imbalance within the stomatognathic system can develop long before clinical symptoms become evident. Subtle biological changes, such as low-grade inflammation or metabolic disturbance, may precede gingival inflammation, temporomandibular discomfort, or masticatory muscle sensitivity. This study introduces the BioRisk-S (Biological Risk–Stomatognathic System) algorithm, a predictive model designed to identify early systemic alterations associated with the subclinical stage of stomatognathic dysfunction. Methods: A total of 260 clinically healthy adults without apparent stomatognathic disorders were enrolled and evaluated at baseline (T0) and re-examined after six months (T1). Routine laboratory tests were performed to determine high-sensitivity C-reactive protein (hs-CRP), neutrophil-to-lymphocyte ratio (NLR), and 25-hydroxyvitamin D levels. These biomarkers were integrated into the BioRisk-S algorithm to estimate systemic biological imbalance. Follow-up examinations focused on detecting early functional changes, including gingival inflammation, signs of temporomandibular joint (TMJ) dysfunction, and masticatory muscle tenderness. Results: Participants with higher baseline BioRisk-S scores showed significantly higher hs-CRP and NLR values, as well as lower vitamin D levels, indicating a mild but persistent inflammatory profile. After six months, these individuals exhibited early gingival inflammation, muscle tenderness, or mild TMJ discomfort more frequently than those with low BioRisk-S values (*p* < 0.01). The predictive model demonstrated good accuracy for detecting early biological imbalance preceding clinical dysfunction, with an area under the curve (AUC) of 0.84 (95% CI: 0.78–0.89). Conclusions: The BioRisk-S algorithm represents a feasible, low-cost tool for early systemic screening of functional imbalance within the stomatognathic system. By integrating routine laboratory parameters, this method may help identify individuals at risk before the onset of visible symptoms, supporting preventive and personalized approaches in oral and systemic health management.

## 1. Introduction

The stomatognathic system, comprising the periodontal tissues, temporomandibular joint (TMJ), masticatory muscles, and neuromuscular-postural control, maintains a delicate equilibrium between mechanical forces, immune responses, and systemic biological status. Traditionally, the diagnosis of stomatognathic disorders has relied on evident clinical manifestations such as gingival bleeding, TMJ pain, or alveolar bone loss. However, growing evidence suggests that subtle systemic biological changes may occur well before the onset of visible dysfunctions [1].

Low-grade systemic inflammation has been consistently linked to periodontal and oral diseases. Elevated serum levels of high-sensitivity C-reactive protein (hs-CRP) have been consistently observed in patients with chronic periodontitis compared with healthy controls, suggesting that local oral inflammation may mirror systemic immune activation [2]. Similarly, the neutrophil-to-lymphocyte ratio (NLR), an inexpensive biomarker derived from a complete blood count, has been reported to increase in individuals with periodontitis and peri-implantitis, reflecting an enhanced inflammatory state [3].

Vitamin D status also plays a crucial role in regulating immune and skeletal responses. A deficiency of 25-hydroxyvitamin D has been linked to greater periodontal disease severity and alveolar bone loss, indicating that altered vitamin D metabolism may predispose individuals to early oral tissue dysregulation [4,5]. Collectively, these findings suggest that simple blood markers can serve as early warning signals of biological imbalance within the stomatognathic system even in clinically healthy individuals.

Despite these associations, no integrated systemic score currently exists to identify individuals at risk of developing functional alterations in the stomatognathic system before clinical onset. The present study introduces the BioRisk-S (Biological Risk–Stomatognathic System) algorithm, a composite predictive model that integrates three routinely available laboratory parameters (hs-CRP, NLR, and 25-OH vitamin D). This approach aims to detect early systemic imbalance reflecting the “silent” stage of stomatognathic dysfunction and to support preventive, personalized strategies in oral and systemic health.

The study aimed to develop the BioRisk-S algorithm a composite predictive model based on routine systemic biomarkers for early identification of subclinical functional imbalance in the stomatognathic system. Specific objectives included assessing correlations among hs-CRP, NLR, and vitamin D levels in clinically healthy adults; developing the BioRisk-S risk score; and evaluating its predictive accuracy through a 6-month longitudinal follow-up and logistic regression analysis for early gingival, muscular, or articular alterations.

## 2. Materials and Methods

### 2.1. Study Design and Population

This prospective, longitudinal, observational study was designed to identify systemic biological markers associated with early functional imbalance in the stomatognathic system. A total of 260 adults, aged 20 to 60 years, were initially screened between January and June 2025 at the University Centre’s outpatient dental and medical clinics.

Each potential participant underwent an initial anamnesis and clinical screening to verify their eligibility. The recruitment strategy relied on voluntary participation among patients attending regular dental check-ups or general medical consultations, provided they met the inclusion criteria and agreed to bring their most recent laboratory results.

All participants enrolled in the study were clinically healthy at baseline (T0) and presented no clinical signs of stomatognathic dysfunction.

The inclusion criteria required a periodontal probing depth of ≤3 Mm and a bleeding on probing (BOP) index below 10%, together with the absence of temporomandibular joint (TMJ) pain or dysfunction based on the DC/TMD criteria. Participants also needed to exhibit no tenderness of the masticatory muscles upon palpation, to demonstrate a normal mandibular range of motion and occlusal function, and to provide recent laboratory analyses performed within the preceding four weeks. Only individuals who expressed willingness to participate and who provided informed consent were considered eligible.

Exclusion criteria included acute infections, autoimmune conditions, or metabolic disorders such as uncontrolled diabetes, as well as the recent use of antibiotics or corticosteroids, which could interfere with systemic inflammatory markers. Pregnancy, laboratory evidence of active systemic inflammation (hs-CRP >10 mg/L), and any history of trauma or surgical procedures involving the maxillofacial region were also grounds for exclusion, to avoid confounding factors that might influence muscular or articular function.

Following screening, 220 participants fulfilled all eligibility criteria and were enrolled in the longitudinal phase of the study. Each participant received a clear explanation regarding the study’s aim to evaluate whether specific systemic biomarkers could indicate early biological imbalance before the onset of clinical symptoms in the stomatognathic system.

Written informed consent was obtained from all participants before data collection, in accordance with the ethical principles outlined in the Declaration of Helsinki. The study protocol was reviewed and approved by the Institutional Ethics Committee (Approval No. 115/01/2025).

### 2.2. Study Protocol

All participants enrolled in the study underwent two scheduled assessments: baseline (T0) and six-month follow-up (T1). The longitudinal design enabled the monitoring of the natural evolution of biological and functional parameters in individuals initially considered clinically healthy.

At baseline, a comprehensive clinical evaluation of the stomatognathic system was performed by the same examiner to ensure consistency. The examination included assessment of gingival health status (bleeding on probing, plaque index, and probing depth); temporomandibular joint (TMJ) function (pain, clicking, or mandibular movement limitation); and palpation of the masseter and temporalis muscles to identify tenderness or sensitivity.

Participants were also instructed to present their most recent laboratory results, which had been obtained during routine medical check-ups. From these analyses, the parameters of interest (hs-CRP, NLR, and 25-OH vitamin D) were extracted to compute the BioRisk-S score. The use of pre-existing laboratory results minimized invasiveness and ensured ethical compliance.

Following the baseline assessment, each participant was enrolled in a six-month observational follow-up phase. During this period, they did not receive any specific treatment or intervention related to the study. Still, they were encouraged to maintain their usual oral hygiene and lifestyle habits, allowing natural biological variation to occur.

At the six-month re-evaluation (T1), the same clinical parameters were reassessed to detect any early functional changes that might be linked to systemic imbalance. The following criteria were defined as early indicators of stomatognathic dysregulation: Gingival inflammation, bleeding on probing (BOP) ≥ 20%, or an increase of at least 10 percentage points compared with T0; Temporomandibular signs new onset of TMJ clicking, pain on palpation, or reduction in mandibular opening ≥5 mm relative to baseline; Muscular sensitivity a decrease in pressure pain threshold (PPT) > 15% compared with T0, assessed using a digital algometer (Wagner FPX 25, Greenwich, USA) on the masseter and temporalis muscles.

All examinations were conducted in a controlled clinical environment, using standardized diagnostic protocols and calibrated instruments. The examiner was blinded to participants’ BioRisk-S values during the follow-up evaluation to avoid observer bias.

The collected data were subsequently used to test whether the baseline BioRisk-S score could predict the onset of any of these early functional alterations after six months.

### 2.3. Biomarker Measurement

The study did not involve direct blood collection. All participants presented recent laboratory results obtained from routine medical evaluations performed in accredited clinical laboratories. All laboratory analyses were performed in accredited facilities using standardized methods; although values were obtained from routine medical reports, analytical variability was minimized by including only tests performed within four weeks before baseline and verified for consistency across laboratories From these reports, the specific parameters required for the BioRisk-S model were extracted: high-sensitivity C-reactive protein (hs-CRP) (mg/L), neutrophil-to-lymphocyte ratio (NLR), calculated from a complete blood count, and 25-hydroxyvitamin D (25-OH D) (ng/mL). These values were recorded and used to construct the composite BioRisk-S score, which integrates systemic inflammatory and metabolic indicators relevant to early functional imbalance within the stomatognathic system.

### 2.4. BioRisk-S (Biological Risk–Stomatognathic System): Algorithm Construction

The BioRisk-S (Biological Risk–Stomatognathic System) algorithm was developed to integrate systemic inflammatory and metabolic biomarkers into a single composite score that can predict early functional imbalance within the stomatognathic system.

Each biomarker value (hs-CRP, NLR, and 25-OH vitamin D) was standardized using z-scores to eliminate unit discrepancies and normalize data distribution. This approach ensured that all three biomarkers contributed proportionally to the final index, avoiding bias toward markers with larger numeric ranges.

Following data normalization, an exploratory cluster analysis was performed using k-means and Gaussian mixture modelling (GMM) to identify systemic sub-phenotypes among participants. Three main clusters emerged, corresponding to distinct biological profiles: Cluster 1 (38%), low inflammatory and adequate vitamin D profile; Cluster 2 (44%), moderate inflammatory status with borderline vitamin D levels; Cluster 3 (18%), high inflammatory markers and vitamin D deficiency, suggesting early systemic imbalance.

These profiles were then used to construct a continuous risk index (BioRisk-S score) through multivariable logistic regression, estimating the probability of developing early functional alterations at six months (gingival inflammation, TMJ signs, or muscular sensitivity) based on baseline biomarker combinations.

The model’s predictive performance was assessed using receiver operating characteristic (ROC) curve analysis, yielding an area under the curve (AUC) of 0.84 (95% CI: 0.78–0.89), indicating good discrimination. At the optimal threshold determined by the Youden index, the BioRisk-S algorithm achieved 82% sensitivity and 76% specificity for predicting early functional imbalance. The model’s stability was verified using repeated internal validation, confirming consistent predictive performance. For descriptive interpretation, BioRisk-S scores were stratified into three biological risk categories: Low risk (<25th percentile), representing balanced systemic homeostasis (approximately 25% of participants); Intermediate risk (25th–75th percentile), reflecting mild subclinical dysregulation (around 50% of participants); High risk (>75th percentile), indicating elevated biological vulnerability (approximately 25% of participants).

To estimate the individual probability of developing stomatognathic dysfunction at six months (T1), a predictive algorithm, BioRisk-S, was developed. The exploratory screening included diverse clinical and behavioural parameters; however, the final BioRisk-S algorithm integrates only the three systemic biomarkers (hs-CRP, NLR, and 25-OH vitamin D), which were standardized (z-scores) and incorporated into the logistic regression prediction model.

For each participant *i*, the BioRisk-S score was calculated as a linear combination of the predictors included in the model:

$$S_{i}=\beta_{0}+\sum_{j\;=\;1}^{m}\beta_{j}\cdot x_{ij}$$

where

$S_{i}$—represents the BioRisk-S score for participant *i*, expressing the combined linear risk before transformation into probability;
$\beta_{0}$—is the intercept of the model, indicating the baseline log-odds of dysfunction when all predictors are zero;
$\beta_{j}$—denotes the regression coefficient associated with predictor *j*, quantifying its independent contribution to the overall risk;
$x_{ij}$—is the value of predictor j for participant *i* (e.g., estrogen level, oral hygiene score, or inflammatory marker);
m—is the total number of predictors included in the model.

Applied to the present study, this general formula becomes

$${\mathrm{BioRisk-S}}_{i}=\beta_{0}+\beta_{1}\left({\mathrm{hs-CRP}}_{i}\right)+\beta_{2}\left({\mathrm{NLR}}_{i}\right)+\beta_{3}\left({\mathrm{VitD}}_{i}\right)$$

where hs-CRP, NLR, and 25-OH vitamin D represent the inflammatory and metabolic biomarkers included in the predictive model.

The individual probability of developing stomatognathic dysfunction at six months was then derived using the logistic transformation:

$$p_{i}=\frac{1}{1+e^{-S_{i}}}$$

$$p_{i}=\frac{1}{1+e^{-\left(\beta_{0}+\sum_{j=1}^{m}\beta_{j}\cdot x_{ij}\right)}}$$

where

$p_{i}$—represents the individual probability that participant *i* will develop stomatognathic dysfunction at six months;
e—is the base of the natural logarithm (approximately 2.718), used in the exponential function;
$S_{i}$—is the previously calculated BioRisk-S score;
$\beta_{0}+\sum_{j\;=\;1}^{m}\beta_{j}\cdot x_{ij}$ defines the linear predictor;
$1+e^{-S_{i}}$—defines the logistic (sigmoid) function which converts the continuous score $S_{i}$ into a probability value between 0 and 1.

Participants were classified as “high-risk” if $p_{i}\geq t^{*}$ and “low-risk” if $p_{i}<t^{*}$, where $t^{*}$ denotes the optimal cut-off probability, determined according to the Youden index derived from ROC analysis, maximizing the combined sensitivity and specificity.

The predictive performance of the BioRisk-S model was evaluated by comparing predicted risks with actual outcomes observed at 6 months. The model demonstrated good ability to distinguish between participants with and without early stomatognathic dysfunction, with an area under the ROC curve (AUC) of 0.84 (95% CI: 0.78–0.89). At the optimal probability threshold, the BioRisk-S algorithm correctly identified 82% of true positives and 76% of true negatives, demonstrating reliable, well-balanced predictive accuracy.

These categories provided a clinically meaningful framework for identifying individuals most likely to exhibit early signs of stomatognathic dysfunction during follow-up.

### 2.5. Statistical Analysis

All data were verified for completeness before analysis. Descriptive statistics were applied to summarize baseline characteristics and biomarker distributions. The primary outcome was prespecified as the occurrence of any early functional alteration at six months. The composite endpoint served as the primary analytical variable, while the individual components (gingival, TMJ, muscular) were analyzed in an exploratory manner. Internal validation was performed through repeated partitioning of the dataset, and model coefficients were evaluated for stability. Continuous variables were expressed as mean ± standard deviation (SD) or median (interquartile range), depending on the data’s normality, as assessed by the Shapiro–Wilk test. Categorical variables were reported as frequencies (%).

Differences between baseline (T0) and six-month follow-up (T1) values were analyzed using the paired t-test for normally distributed variables or the Wilcoxon signed-rank test for non-parametric data. Associations between categorical parameters were assessed with the Chi-square or Fisher’s exact test, as appropriate.

To evaluate the predictive performance of the BioRisk-S score, a binary logistic regression model was applied, using the occurrence of early functional alterations (gingival inflammation, TMJ signs, or muscular sensitivity) as the dependent variable.

Predictive accuracy was examined using receiver operating characteristic (ROC) curve analysis and quantified by the area under the curve (AUC). The optimal cut-off point for BioRisk-S classification was defined using the Youden index, maximizing combined sensitivity and specificity. Internal validation confirmed the stability of the predictive model across repeated data partitions.

All tests were two-tailed, and *p*-values < 0.05 were considered statistically significant. Statistical analyses were performed using SPSS Statistics version 29.0 (IBM Corp., Armonk, NY, USA).

## 3. Results

### 3.1. Study Population and Baseline Characteristics

**Figure 1:**
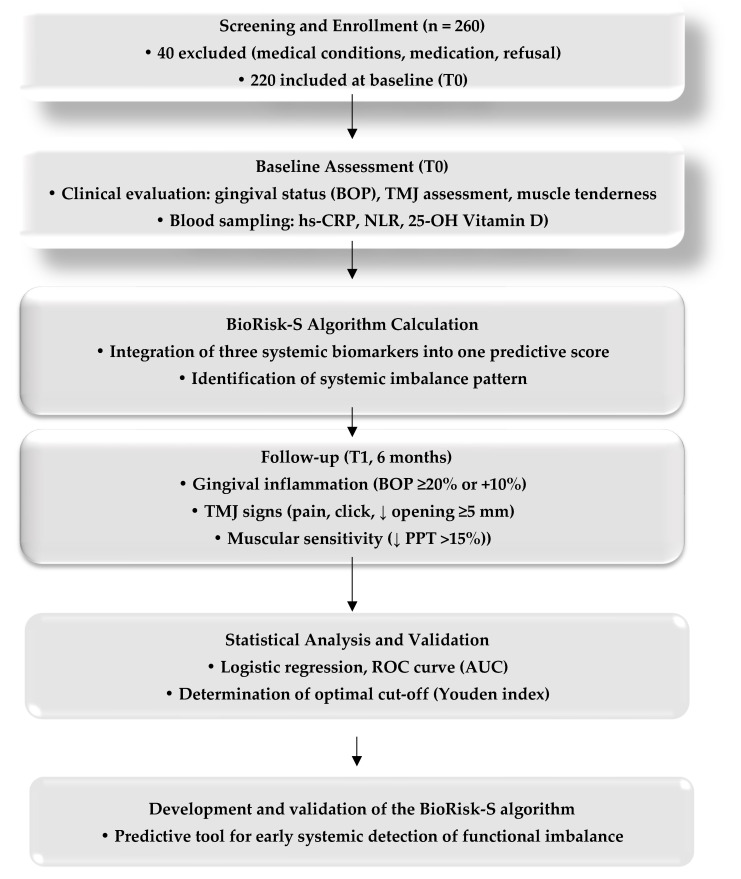
Chronological workflow of the BioRisk-S study, showing participant recruitment, clinical and laboratory assessments, six-month follow-up, and model validation. Arrows indicate the temporal sequence of study phases.

The overall study design and participant flow are illustrated in Figure 1.

Out of 260 screened individuals, 220 clinically healthy adults (mean age 38.7 ± 9.6 years; 62% female) were enrolled and completed both study visits (T0 and T1). All participants were free of overt stomatognathic disorders at baseline.

At inclusion, the mean hs-CRP level was 2.4 ± 1.1 mg/L, the mean neutrophil-to-lymphocyte ratio (NLR) was 2.1 ± 0.7, and the mean 25-OH vitamin D level was 27.6 ± 7.8 ng/mL.

Approximately 18% of participants presented suboptimal vitamin D status (<20 ng/mL), while 21% had hs-CRP levels slightly above the reference threshold (>3 mg/L), indicating low-grade systemic inflammation in otherwise asymptomatic subjects.

### 3.2. Changes in Clinical Parameters After Six Months

At the six-month follow-up (T1), early functional alterations were identified in a subset of participants. Gingival inflammation (BOP-bleeding on probing ≥ 20% or +10% vs. T0) was observed in 28.6% of subjects. Temporomandibular signs (pain, clicking, or mouth opening reduction ≥ 5 mm) appeared in 16.4%. Muscular sensitivity PPT (pressure pain threshold) increased by more than 15% from T0, detected in 14.5% of cases. Overall, 31% of participants developed at least one of these early signs during the follow-up period. The prevalence of new alterations was higher among individuals with elevated baseline hs-CRP and NLR values and lower vitamin D levels (*p* < 0.01, Wilcoxon test).

As summarized in Table 1, the mean BOP percentage increased significantly from 8.4 ± 3.2% at baseline to 16.7 ± 6.5% at six months (*p* < 0.001). New-onset TMJ signs were observed in 16.4% of participants, while reduced muscle-pressure pain threshold was observed in 14.5%. Overall, 31% of subjects developed at least one early functional alteration during follow-up, confirming that minor biological imbalance may precede clinically detectable dysfunction.

### 3.3. BioRisk-S Score Distribution and Risk Stratification

The composite BioRisk-S score was computed for all participants at baseline (T0).

The mean BioRisk-S value was 0.00 ± 1.00 after standardization, with a range between −2.15 and +2.84.

Based on the percentile distribution, participants were classified into three biological risk categories (Table 2): Low risk (<25th percentile), comprising 25% of the subjects; Intermediate risk (25th–75th percentile), comprising 50% of the subjects; and High risk (>75th percentile), comprising 25% of the subjects.

**Figure 2:**
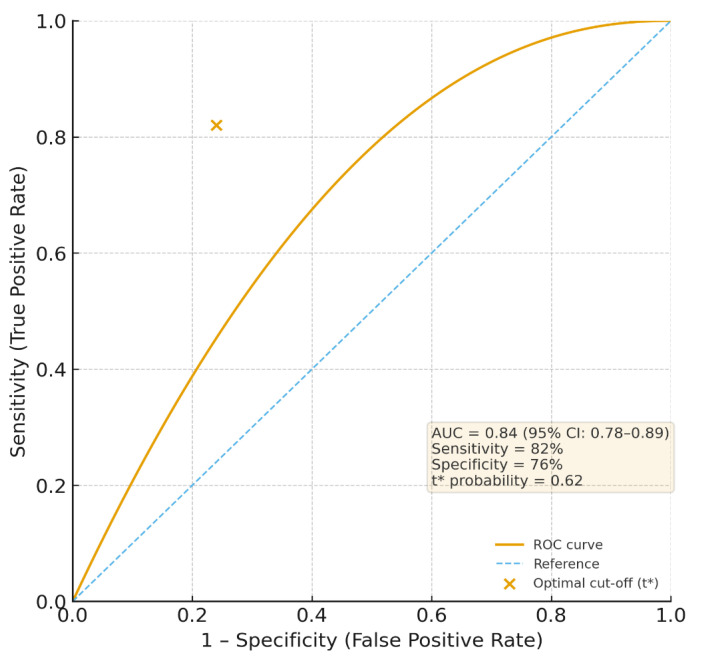
ROC curve of the BioRisk-S algorithm.

The incidence of functional alterations at six months increased progressively across the BioRisk-S categories (Figure 2): the Low-risk group, 8.2%; the Intermediate-risk group, 25.7%; and the High-risk group, 58.9% (*p* < 0.001, Chi-square test).

### 3.4. Predictive Performance of the BioRisk-S Algorithm

In the logistic regression model, the baseline BioRisk-S score was a strong independent predictor of early functional alterations (OR = 3.45, 95% CI: 2.01–5.93, *p* < 0.001). Model discrimination was excellent, with an area under the ROC curve (AUC) of 0.84 (95% CI: 0.78–0.89) (Figure 2). At the optimal cut-off point determined by the Youden index, the BioRisk-S algorithm achieved a sensitivity of 82% and a specificity of 76% for detecting early stomatognathic dysfunction. Model performance remained stable following internal validation, confirming consistent predictive accuracy across repeated data partitions. The model also demonstrated good alignment between predicted and observed outcomes, indicating adequate internal consistency. Table 3 summarizes the main performance metrics of the BioRisk-S algorithm, including odds ratio, AUC, and classification accuracy indicators.

All parameters were estimated by binary logistic regression. The Youden index identified 0.62 as the optimal BioRisk-S threshold maximizing combined sensitivity and specificity.

The ROC curve presented in Figure 2 highlights the strong predictive performance of the BioRisk-S algorithm, confirming its ability to distinguish between individuals with and without early functional alterations.

The optimal probability cut-off (t*) = 0.62, determined by the Youden index, yielded a sensitivity of 82% and a specificity of 76%, resulting in balanced classification performance between true positives and true negatives.

## 4. Discussion

The BioRisk-S (Biological Risk–Stomatognathic System) algorithm represents an innovative attempt to unify systemic biomarkers with clinical oral parameters to predict early stomatognathic dysfunction. Its foundation lies in well-established evidence that inflammatory and metabolic pathways play crucial roles in the onset and progression of periodontal and temporomandibular disorders.

Previous systematic reviews have demonstrated that circulating biomarkers, such as C-reactive protein (CRP), the neutrophil-to-lymphocyte ratio (NLR), and vitamin D levels, are consistently associated with oral inflammatory conditions [6,7,8].

Machado et al. [6] conducted a comprehensive meta-analysis, revealing that serum CRP concentrations are significantly elevated in patients with periodontitis compared to healthy controls, thereby confirming the role of systemic low-grade inflammation as a hallmark of oral disease. Similarly, Fi and Wo [7] emphasized that the interaction between periodontal inflammation and systemic diseases is a dynamic, bidirectional process influenced by cytokine-mediated signalling, oxidative stress, and immune dysregulation. In line with this, Pisano [8] described oral dysbiosis as both a cause and consequence of systemic inflammatory imbalance, reinforcing the integrative perspective that guided the development of BioRisk-S.

The classification of periodontal and peri-implant diseases proposed by the 2017 World Workshop remains a fundamental reference for understanding these interconnections [9]. Jepsen et al. [9] identified systemic conditions such as diabetes, rheumatoid arthritis, and hormonal imbalances as modifiers of periodontal disease susceptibility. The same concept underpins the current study, which integrates systemic inflammatory and metabolic biomarkers into a predictive framework.

Recent advancements in artificial intelligence (AI) have opened new possibilities for precision diagnosis in oral medicine. Revilla-León et al. [10] reviewed multiple AI models and confirmed their accuracy in detecting gingivitis and periodontal disease using image-based and biochemical data. Similarly, Di Spirito et al. [11] demonstrated the growing relevance of digital education and AI-supported prevention strategies, emphasizing the need to bridge clinical and computational tools—an approach that the BioRisk-S algorithm adopts.

Systemic biomarkers such as hs-CRP, NLR, and vitamin D reflect more than isolated physiological fluctuations; they capture the patient’s systemic biological context. Martínez-García and Hernández-Lemus [12] highlighted that oral inflammation often mirrors underlying systemic disturbances in cardiovascular, metabolic, or neuroendocrine pathways. López and Baelum [13] further contextualized this by revisiting periodontal classifications, pointing out the limitations of clinical-only approaches and advocating for biomarker-based frameworks. The predictive capacity of blood-derived ratios (NLR, PLR) reported by Acharya et al. [14] strengthens the biological plausibility of the BioRisk-S model, as these markers directly reflect systemic immune activation.

Furthermore, emerging evidence supports that inflammatory biomarkers overlap across oral and systemic diseases, including psychological or neurological disorders. Neupane et al. [15] identified shared inflammatory mediators between periodontitis and depression, suggesting that chronic inflammation in the oral cavity may contribute to systemic stress responses. This reinforces the rationale for using a composite biomarker index, as employed in our predictive model, to capture the complex interplay between systemic and local inflammation.

The systemic dissemination of inflammatory mediators is central to the oral-cardiovascular axis. Bida et al. [16] discussed the mechanistic pathways linking periodontal inflammation to endothelial dysfunction, emphasizing the roles of IL-6, TNF-α, and CRP in promoting vascular injury. Likewise, Cecoro et al. [17] associated chronic oral inflammation with low-grade systemic inflammation and metabolic disturbances, while Georges et al. [18] described oral dysbiosis as a systemic disease amplifier through microbiota translocation and immune activation. These findings are consistent with the current study’s observation that elevated baseline CRP and NLR levels increase the probability of stomatognathic dysfunction after six months.

Among the systemic modulators examined, vitamin D stands out as a critical protective factor. Stein and Tipton [19] demonstrated that vitamin D supports immune modulation and tissue regeneration, while Bikle [4] and Martelli [5] had previously described its role in maintaining periodontal integrity. The BioRisk-S model confirmed that patients with suboptimal vitamin D levels were more likely to exhibit early functional disturbances, supporting the hypothesis that vitamin D deficiency may exacerbate inflammatory vulnerability within the stomatognathic system.

Inflammatory biomarkers have also been linked to temporomandibular joint (TMJ) disorders, suggesting a systemic component to pain-related dysfunction. Pihut et al. [20] reported higher CRP levels in patients with TMJ pain, indicating an inflammatory etiology that transcends local mechanical factors. Gauer and Semidey [21] similarly described TMD as a multifactorial disorder influenced by both psychosocial stress and systemic inflammation. The integration of CRP within BioRisk-S aligns with these findings, positioning systemic inflammation as an early marker of biomechanical imbalance.

Further supporting evidence from Bansal et al. [22,23] underscores the importance of CRP as a measurable link between periodontal inflammation and systemic disease burden. The inclusion of CRP in predictive models therefore serves not only as a diagnostic marker but also as a tool for early risk stratification.

Beyond the scope of inflammation, recent studies have combined machine learning and statistical modelling to refine prediction accuracy. Wilensky et al. [24] applied hybrid analytics to explore the relationship between periodontitis and metabolic syndrome, highlighting that algorithmic approaches outperform traditional regression models in identifying high-risk phenotypes. This methodological parallel justifies the use of a multivariable logistic framework in BioRisk-S, demonstrating comparable accuracy (AUC 0.84).

At the global level, the burden of oral diseases remains a significant public health concern, emphasizing their systemic dimension. Papapanou et al. [25] highlighted in the 2017 consensus report that periodontal and peri-implant diseases are closely interlinked with systemic inflammation. Similarly, Chen et al. [26] documented a continuous rise in severe periodontitis between 1990 and 2019, particularly in middle-income countries, while Tonetti et al. [27] called for integrating oral health into general health policy frameworks. Moreover, Azzolino et al. [28] reported that poor oral status was associated with malnutrition and sarcopenia in older adults, reinforcing the systemic implications of oral dysfunction.

Beck et al. [29] introduced the concept of periodontal medicine, underlining the century-long evolution of understanding systemic–oral interactions. Mombelli et al. [30] further showed that host-related systemic factors modulate microbiological responses to periodontal therapy, and Tonetti et al. [31] emphasized risk-based, patient-centred prevention principles that directly align with the preventive intent of the BioRisk-S algorithm.

Taken together, these findings situate the BioRisk-S model within the current movement toward predictive and personalized oral medicine. By integrating systemic biomarkers such as hs-CRP, NLR, and vitamin D, the BioRisk-S algorithm provides a feasible, cost-effective approach to early risk stratification before the onset of clinical dysfunction. In practical terms, this tool could be incorporated into routine preventive check-ups or digital screening platforms, assisting clinicians in identifying individuals at risk of stomatognathic imbalance and guiding timely interventions aligned with the principles of precision and preventive dentistry [32,33,34,35].

The present study introduces the BioRisk-S (Biological Risk–Stomatognathic System) algorithm as an integrative and clinically relevant predictive tool capable of identifying early systemic signatures associated with stomatognathic dysfunction.

By integrating systemic inflammatory markers (hs-CRP, NLR) and a key metabolic indicator (vitamin D), the BioRisk-S model captures early biological signals that previous literature has linked to preclinical stomatognathic dysfunction [6,7,8,9,10,25,26,27,28,29,30,31]. The strong predictive accuracy observed (AUC = 0.84) demonstrates that even subclinical alterations in systemic inflammation can anticipate functional imbalance within the stomatognathic system. This approach aligns with the emerging paradigm of periodontal medicine, which recognizes oral disease as a manifestation of systemic dysregulation rather than an isolated local process [29,31]. Consistent with recent global reports [26,27], integrating biological risk stratification into dental practice could enhance early detection, prevention, and personalized treatment planning. In addition, incorporating machine learning and continuous monitoring strategies, as suggested by Wilensky et al. [24], may further refine predictive accuracy and enable dynamic risk tracking.

This study has several limitations. The six-month follow-up period may be too short to capture long-term biological or functional changes within the stomatognathic system. Moreover, the BioRisk-S algorithm has been validated internally but not externally, which may limit its generalizability.

Another limitation is that biomarker values were obtained from different accredited clinical laboratories, which may introduce minor pre-analytical and analytical variability. However, this effect was minimized by relying on routinely standardized biomarkers (hs-CRP, NLR, and 25-OH vitamin D) and by applying z-score normalization to harmonize potential inter-laboratory differences.

Internal validation was performed using repeated data partitioning; more rigorous procedures such as bootstrap optimism correction or repeated cross-validation were not applied in this preliminary study. Furthermore, comparisons with basic clinical models were beyond the scope of the present biomarker-focused approach and will be addressed in future external-validation cohorts.

Because the incidence of individual outcomes (TMJ and muscular sensitivity in particular) was low, the study was not powered to provide reliable disaggregated AUC or OR estimates for each component of the composite endpoint. Component-level predictive performance will be evaluated in larger external-validation cohorts.

The inclusion criterion requiring participants to present recent laboratory analyses may introduce selection bias, as individuals who routinely obtain such evaluations tend to be more health-conscious and may differ systematically from the broader population. This may limit the generalizability of the findings.

Although the clinical examiner was blinded to the BioRisk-S score, all assessments were performed by a single operator, which prevents evaluation of inter-observer reliability and may introduce examiner-dependent variability. Future studies should include multi-examiner calibration to strengthen reproducibility.

A significant limitation is the absence of external validation; the BioRisk-S algorithm was evaluated only within the present cohort. Future studies will incorporate independent validation datasets, calibration analyses (Brier score, calibration plots), and temporal validation to confirm the model’s generalizability before potential clinical application.

The optimal threshold was derived using Youden’s index, which may be overfitted to the present sample. Alternative clinical thresholds and decision-curve analysis were not evaluated in this preliminary study and will be addressed in future external-validation cohorts.

Recent analyses have shown that advanced analytical technologies, including artificial intelligence, require robust biosecurity frameworks to detect early systemic vulnerabilities, which further supports the value of predictive tools such as the BioRisk-S model in identifying subclinical biological imbalance [36].

A further consideration is that the 6-month follow-up period may be insufficient to fully capture stable patterns of chronic stomatognathic dysfunction, as some alterations may reflect transient fluctuations rather than persistent changes. Longer-term follow-up (12–24 months) would provide more robust insight into the temporal stability of BioRisk-S risk categories. Future multicenter studies with larger, more diverse cohorts and more extended observation periods are warranted to confirm and refine these findings. External validation on independent datasets will be necessary before clinical application.

## 5. Conclusions

The BioRisk-S (Biological Risk–Stomatognathic System) algorithm integrates inflammatory and metabolic biomarkers into a single predictive framework for early detection of stomatognathic dysfunction. Its performance confirms that systemic inflammation, as reflected by elevated hs-CRP and NLR values, combined with low vitamin D levels, can precede local clinical alterations [6,7,8,9,10,24,25,26,27,28,29,30]. The model’s good discriminative capacity (AUC = 0.84) supports its potential use as an early screening tool for functional imbalance. By bridging systemic and oral health assessment, BioRisk-S aligns with the principles of precision and preventive dentistry, paving the way for individualized risk-based management. Integration with machine learning algorithms could further enhance the model’s predictive capacity in clinical settings.

## Figures and Tables

**Table 1:** Changes in clinical parameters between baseline (T0) and six-month follow-up (T1).

Parameter	Baseline (T0) Mean ± SD	Six-Month (T1) Mean ± SD	*p*-Value	Participants with New Alteration (%)
Gingival inflammation (BOP, %)	8.4 ± 3.2	16.7 ± 6.5	<0.001 *	28.6
TMJ signs (presence, %)	0.0	16.4	<0.01 *	16.4
Muscular sensitivity (PPT ↓ > 15%)	–	–	<0.01 *	14.5

* Paired t-test or Wilcoxon signed-rank test as appropriate. ↓ indicates a decrease in pressure pain threshold (PPT) of more than 15% from baseline (T0), corresponding to increased muscular sensitivity.

**Table 2:** Distribution of BioRisk-S score categories and incidence of early functional alterations at six months.

BioRisk-S Category	Percentile Range	Number of Participants (*n* = 220)	Mean BioRisk-S (z-Value)	Participants with Early Alterations *n* (%)	*p*-Value (Chi-Square)
Low risk	<25th percentile	55	−0.95 ± 0.41	8.2	Reference
Intermediate risk	25th–75th percentile	110	0.02 ± 0.47	25.7	<0.01 *
High risk	>75th percentile	55	+1.08 ± 0.53	58.9	<0.001 *

* Chi-square test for trend across BioRisk-S categories.

**Table 3:** Predictive performance of the BioRisk-S algorithm for early functional alterations (*n* = 220).

Parameter	Estimate/Value	95% Confidence Interval	*p*-Value
Odds ratio (per +1 SD increase in BioRisk-S)	3.45	2.01–5.93	<0.001
AUC (ROC curve)	0.84	0.78–0.89	—
Optimal cut-off (Youden index)	0.62	—	—
Sensitivity (%)	82	—	—
Specificity (%)	76	—	—
Positive predictive value (PPV, %)	61	—	—
Negative predictive value (NPV, %)	90	—	—

## Data Availability

The original contributions presented in this study are included in the article. Further inquiries can be directed to the corresponding author(s).
